# The Case of Spinal Curvature

**Published:** 1886-11-13

**Authors:** 


### Difficult Cases.

*The intention of this column is to afford assistance to clergymen, district visitors, hospital officials, medical men, and others who have to provide for the treatment or accommodation of difficult cases of disease, sometimes in its acute, but usually in its chronic form. Anyone being in doubt as to the best course to pursue in any such case, is invited to communicate with the Editor. All such communications should be written on one side of the paper only, and be addressed to the Editor, the envelope being marked in the top left-hand corner, "Difficult Cases."*

### The Case of Spinal Curvature.

We have made inquiries as to the case referred to by "A Colonel's Wife," and have now the pleasure to announce that, through the kindness of Mr. W. H. A. Jacobson, F.R.C.S., and the assistance of Mr. Custance, the Secretary of the Metropolitan Hospital Sunday Fund, the child will be received for several weeks' treatment into the Royal Hospital for Women and Children, in the Waterloo Bridge Road. At the expiration of this time the case will not be lost sight of, and if further aid is considered necessary or likely to prove beneficial, such additional assistance will be provided. We desire to direct special attention to this case, as it is the aim of the Hospitals Association to help all who may be interested in difficult cases of this kind to find just what they want with the least possible delay and difficulty. If this fact was widely known and acted upon, many poor sufferers would have cause for rejoicing and thankfulness.

Dr. Fell.—Tom Brown, being in disgrace, was set by Dr. Fell, of Christ Church, to translate the thirty-third epigram of Martial:

"Non amo te Zabidi, nec possum dicere quare
Hoc tantum possum dicere, non amo te.

He accomplished his task thus:—

I do not like thee, Dr. Fell—
The reason why I cannot tell;
But this I know, and know full well,
I do not like thee, Dr. Fell.

